# Fexapotide triflutate: results of long-term safety and efficacy trials of a novel injectable therapy for symptomatic prostate enlargement

**DOI:** 10.1007/s00345-018-2185-y

**Published:** 2018-01-29

**Authors:** Neal Shore, Ronald Tutrone, Mitchell Efros, Mohamed Bidair, Barton Wachs, Susan Kalota, Sheldon Freedman, James Bailen, Richard Levin, Stephen Richardson, Jed Kaminetsky, Jeffrey Snyder, Barry Shepard, Kenneth Goldberg, Alan Hay, Steven Gange, Ivan Grunberger

**Affiliations:** 1 Carolina Urologic Research Center, Myrtle Beach, SC USA; 2 Chesapeake Urology Research Associates, Baltimore, MD USA; 3 Accumed Research, Garden City, NY USA; 4 San Diego Clinical Trials, San Diego, CA USA; 5 Atlantic Urology Medical Group, Long Beach, CA USA; 6 Urological Associates of Southern Arizona, Tucson, AZ USA; 7 Freedman Urology, Las Vegas, NV USA; 8 First Urology, Louisville, KY USA; 9 Chesapeake Urology Research Associates, Towson, MD USA; 10 Jean Brown Research, Salt Lake City, UT USA; 11 University Urology, New York, NY USA; 12 Genitourinary Surgical Consultants, Denver, CO USA; 13 Urological Surgeons of Long Island, Garden City, NY USA; 14 U T Southwestern Dept of Urology, Lewisville, TX USA; 15 Willamette Urology, Salem, OR USA; 16 Summit Urology Group, Salt Lake City, UT USA; 17 Brooklyn Urology, Brooklyn, NY USA

**Keywords:** BPH, Urology, LUTS, Fexapotide triflutate, NX-1207

## Abstract

**Purpose:**

These studies were undertaken to determine if fexapotide triflutate 2.5 mg transrectal injectable (FT) has significant long-term (LT) safety and efficacy for the treatment of benign prostatic hyperplasia (BPH).

**Methods:**

Two placebo controlled double-blind randomized parallel group trials with 995 BPH patients at 72 sites treated 3:2 FT:placebo, with open-label FT crossover (CO) re-injection in 2 trials *n* = 344 and long-term follow-up (LF) 2–6.75 years (mean 3.58 years, median 3.67 years; FT re-injection CO mean 4.27 years, median 4.42 years) were evaluated. 12 months post-treatment patients elected no further treatment, approved oral medications, FT, or interventional treatment. Primary endpoint variable was change in Symptom Score (IPSS) at 12 months and at LF. CO primary co-endpoints were 3-year incidence of (1) surgery for BPH in FT treated CO patients versus patients crossed over to oral BPH medications and (2) surgery or acute urinary retention in FT-treated CO placebo patients versus placebo patients crossed over to oral BPH medications. 28 CO secondary endpoints assessed surgical and symptomatic outcomes in FT reinjected patients versus conventional BPH medication CO and control subgroups at 2 and 3 years.

**Results:**

FT injection had no significant safety differences from placebo. LF IPSS change from baseline was higher in FT treated patients compared to placebo (median FT group improvement − 5.2 versus placebo − 3.0, *p* < 0.0001). LF incidence of AUR (1.08% *p* = 0.0058) and prostate cancer (PCa) (1.1% *p* = 0.0116) were both reduced in FT treated patients. LF incidence of intervention for BPH was reduced in the FT group versus oral BPH medications (8.08% versus 27.85% at 3 years, *p* < 0.0001). LF incidence of intervention or AUR in placebo CO group with FT versus placebo CO group with oral medications was reduced (6.07% versus 33.3% at 3 years, *p* < 0.0001). 28/28 secondary efficacy endpoints were reached in LF CO re-injection studies.

**Conclusions:**

FT 2.5 mg is a safe and effective transrectal injectable for LT treatment of BPH. FT treated patients also had reduced need for BPH intervention, and reduced incidence of PCa and AUR.

**Electronic supplementary material:**

The online version of this article (10.1007/s00345-018-2185-y) contains supplementary material, which is available to authorized users.

## Introduction

Benign prostatic hyperplasia begins in middle age and affects the majority of men by age 70–80 [1–7]. BPH leads to chronic lower urinary tract symptoms (LUTS) that may necessitate interventional therapy if the symptoms are deemed excessively bothersome or otherwise are adversely affecting patients’ health status. Prostate enlargement may cause urethral compression, diminished urinary flow, bladder hypertrophy, urinary urgency, frequency, nocturia, straining, urinary tract infections, and urinary retention which can lead to obstructive nephropathy. Management includes diagnostic evaluation to exclude prostate cancer or benign conditions that are not attributable to prostate hyperplasia, treatment options with medications (alpha blockers; 5-alpha reductase inhibitors), and interventional therapies when necessary, such as transurethral minimally invasive surgical treatments (MIST) or invasive surgery (transurethral resection (TURP) or laser ablation) [1–7]. Oral medications and surgical treatments may be associated with unacceptable adverse events. Medications are oftentimes stopped due to poor risk:benefit ratio as well as tachyphylaxis, adherence, cost, and other drug interactions [8–16]. TURP, historically acknowledged as the ‘gold standard’, is effective but associated with anesthetic risk, post-operative morbidities of voiding discomfort, bleeding, incontinence, and potential sexual dysfunction.

FT is a new molecular entity which stimulates caspase pathways (activation of caspases 7, 8, and 10, caspase recruitment domains 6, 11, and 14, and DIABLO), tumor necrosis factor pathways (activation of TNF1, TNFSF6, TNFSF8, TNFSF9, CD70 ligands, and TNFRSF19L, TNFRSF25, TRAF2, TRAF3, TRAF4, TRAF6 receptors), and BCL pathways (activation of BIK, HRK, BCL2L10 and BCL3) in prostate glandular epithelial cells. FT selectively causes loss of cell membrane integrity, mitochondrial metabolic arrest, depletion of RNA, DNA lysis and aggregation, and cell fragmentation and cell loss (Online Figures 2, 3) with subsequent decompression of the urethral lumen.‎ On the basis of evidence from detailed toxicology studies in dogs given injections of FT directly into adjacent structures (bladder, urethra, rectum, periprostatic tissue) the adjacent tissues are unaffected. The underlying safety of FT is based on: 1. selective sparing of critical structures (nerves, periprostatic tissue, bladder, urethra, and rectum) (Online Figure 2); 2. pharmacokinetic (PK) properties which limit extraprostatic exposure; 3. route of administration without a catheterization or significant anesthetic requirement; 4. avoidance of detectable immune response; and 5. an absence of an effect on testosterone or subsequent ejaculatory parameters. FT is administered as an outpatient ultrasound guided transrectal (TR) injection into the transition zone. The procedure time requirement is approximately 3–5 min and does not require a urethral catheter, intravenous or general anesthetic, and apart from a standard TRUS requires no specialized equipment or instrumentation [17, 18].

This report presents the safety and efficacy data from 2 long-term follow-up (LF) prospective double-blind randomized placebo controlled studies and 2 crossover (CO) studies of FT for BPH involving 995 patients at 72 U.S. sites conducted from 2009 to 2017.

## Patients and methods

NX02-0017 and NX02-0018 (0017/0018) were randomized, double-blind, parallel group studies designed to demonstrate safety and efficacy of transrectal ultrasound (TRUS) guided intraprostatic FT 2.5 mg in 10 mL phosphate buffered saline (PBS) sterile solution, compared to placebo 10 mL (vehicle alone), directed to the transition zone (5 mL to each side) with the needle tip and injection as visualized by TRUS [17, 18]. Studies were conducted at 72 U.S. sites (85 U.S. sites approved and initiated; 80 with patient screening and assessments; 72 with patient enrollments) from 2009 to 2017, approved by institutional review boards and FDA (clinicaltrials.gov: NCT00918983, NCT00945490, NCT01438775, NCT01846793). Informed consent was obtained from all individual participants included in the study. Patients were enrolled based on BPH Symptom Score (IPSS), TRUS prostate volume (PV), urinary peak flow rate (Qmax) (Online Table 1). Patients were centrally randomized in a 3:2 ratio FT:placebo by a computer-generated randomization schedule executed by non-study personnel at an independent randomization service provider with no contact except by interactive voice response system. Patients, investigators, all site and non-site study personnel, monitors, and outcome assessors were blinded as to randomization. The following were captured at baseline and 10 days, 1, 3, 6, 9, 12 months: IPSS, Qmax (3, 6, 12 months), PV (12 months), BPH Impact Index (BII), Sexual Function Questionnaire (SFQ), and safety parameters. IPSS, BPH treatments and urological events were prospectively captured at LF. *Crossover studies:* 351 patients after completion of 0017/0018 (maintaining initial treatment double blind) were randomized (*n* = 344 treated) at intervals of 0.5–39.1 months after the first year (mean 20.4 (SD 7.15) months post-randomization) into 2 open-label FT re-injection 6 month studies NX02-0020 and NX02-0022 (0020/0022) with additional LF. 0020/0022 were required for re-injection safety/efficacy data and for patient access to FT after 1 year. Blinded patients could elect no further treatment; oral conventional BPH medications; surgical treatment; or FT treatment. LF pre-specified comparator outcomes included objective measures of incidence of surgery and incidence of AUR, as well as self-reported IPSS and nocturia scores. Safety outcomes (including sepsis, new incidence of prostate cancer, etc.) were pre-planned.

### Statistical methods

The ITT (Intent-to-treat) population included all injected patients with ≥ 1 post-treatment IPSS. Primary Outcome (PO) in 0017/0018 was change in IPSS from baseline to 365 days and at LF. The PO variable did not change throughout the 0017/0018 trials. LF PO data capture did not change and was pre-specified in the 2009 protocols which included the LF questionnaires. These are the first ever adequately powered Phase 3 BPH injectable trials to be fully accrued and completed in the U.S. Therefore, the LF date was not determined until after the 1 year capture was completed (5.0 years after first patient in). The crossover 0020/0022 PO variable did not change during the trial visits (6 months). The 0020/0022 LF extension data capture was pre-specified in the initial protocols (2012) which included LF questionnaires and was unchanged. 0020/0022 LF efficacy PO variables and 28 additional secondary outcome variables were pre-specified initially in the LF Statistical Plan and were unchanged. 0017/0018 were powered with a sample size of 500 subjects (300 FT: 200 Placebo) for PO assuming a 2-sided mean value t test with 0.05 error and 80% power. Secondary endpoints, pre-specified PO subgroups, and additional efficacy endpoints are summarized online. For LF PO analysis all treatment failures were included (worst case scenario analysis). Patients with post-randomization BPH surgery/MIST at anytime were treated as failures (0 change from baseline regardless of any prior reported improvement, or were treated > 0 if last reading prior to withdrawal reported worsening). Patients who withdrew consent due to lack of efficacy, or who refused to participate were treated as treatment failures (0 change from baseline) regardless of prior reported improvement. Patients who subsequently took medications were treated as their last ≥ 0 worsening value unless they had reported improvement (< 0) prior to taking medications in which instances they were excluded. Patients lost to follow-up (LTFU) after multiple documented attempts to reach them, were excluded. Missing values were analyzed according to multiple imputations. *LF outcomes in CO 0020/0022*: Incidence of BPH surgery and AUR within 24 and 36 months post-randomization were compared according to the groups in Table 3 and Online Figure 1. Mean IPSS and nocturia response were compared using *t* tests or non-parametric tests. The Co-POs were 1. Incidence of BPH surgery in 0017/0018 patients with CO FT, compared to 0017/0018 patients who had subsequent conventional BPH medications; 2. Incidence of BPH surgery or AUR in placebo 0017/0018 patients with CO FT, compared to placebo 0017/0018 with subsequent medications. Other group comparisons are in 28 additional Secondary Endpoints (Table 3). Step-down was by closed test procedure.

## Results

**Fig. 1:**
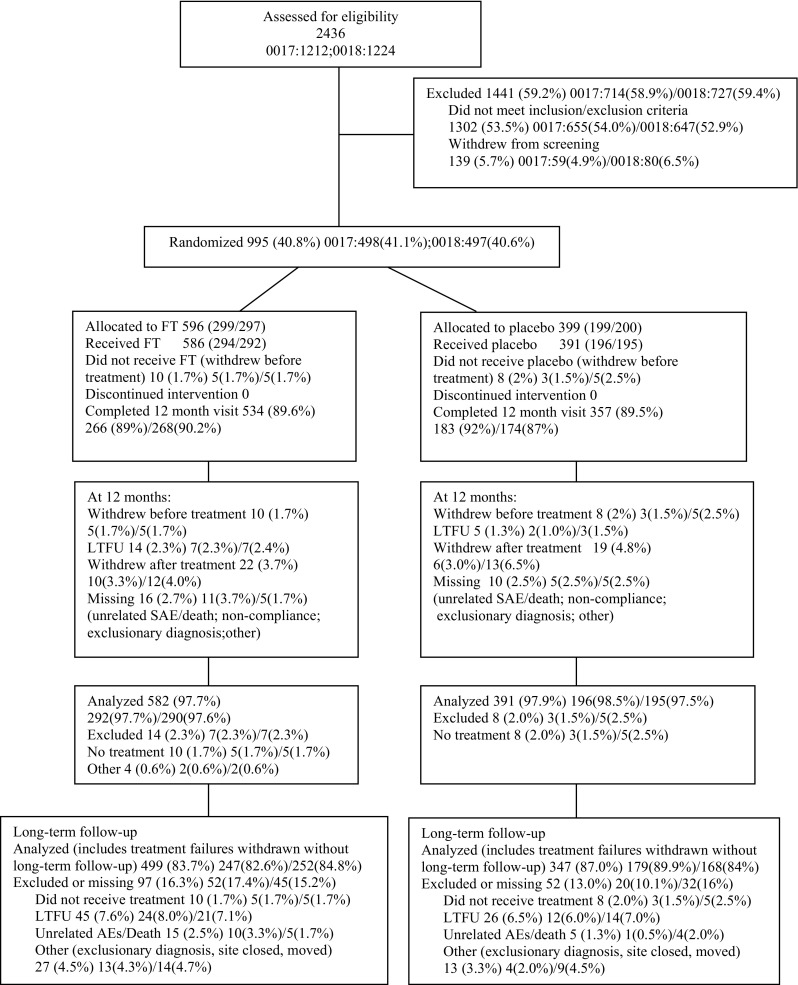
CONSORT diagram of patient enrollment, allocations, treatment and follow-up

0017/0018 enrolled 498 (1212 screened) and 497 (1224 screened) patients, respectively. Baseline characteristics, demographics, and patient disposition are summarized in Online Tables 2, 3, 4 and CONSORT diagram (Fig. 1). ITT lost to follow-up percentages were 1.4–1.6% at 12 months and 7.1–7.3% at LF (mean 43 months). At LF 2.9% of patients had unrelated serious adverse events (SAEs) or death, or otherwise inability to provide a valid questionnaire response (e.g., dementia or other exclusion criteria). LF was 2–6.75 years (mean 3.58 years, median 3.67 years; FT re-injection CO mean 4.27 years, median 4.42 years).

### Safety and side effects

AE data and laboratory results were similar in FT and placebo groups. Reported related AEs in both groups were from prophylactic antibiotics and needle insertion; these AEs were mainly mild, transient and not requiring treatment. Unrelated deaths in the FT group were 0.5% in the first 12 months (2 unrelated myocardial infarctions and 1 pre-existent pulmonary fibrosis), versus 0.25% in placebo (1 unrelated suicide at 9 months). In the reinjection CO studies there were no deaths during the first year post-treatment. Incidence of culture confirmed urinary tract infection (UTI) < 30 days post-treatment was not increased (0.6% in FT and 0.5% in placebo) and there were no cases of urosepsis in FT-treated patients. After 12 months, all statistical analyses of hematological and clinical chemistry parameters showed no changes, and mean serum PSA values were not significantly altered. Semen analyses of a subgroup of *n* = 41 FT samples and *n* = 32 placebo consecutively available samples were overall unchanged from baseline and no FT changes versus placebo were found. Anti-FT antibodies were not detected in *n* = 1072 samples (*n* = 574 FT *n* = 498 controls). PK analyses of *n* = 106 consecutive plasma samples drawn at 1, 5, 10 and 20 min post FT injections showed no extraprostatic systemic signal. After 4 years PCa newly diagnosed after the first 12 months in FT treated (1.1%) was reduced compared to placebo (5.3%) (*p* = 0.0116) (Table 1). Incidence of spontaneous AUR within 3 years in the FT group was reduced (1.08%) compared to placebo population (5.63%) (*p* = 0.0058) (Table 1). There were no transient or persistent FT-related sexual side effects.

**Table 1:** Incidence of prostate cancer and spontaneous AUR. NX02-0017, NX02-0018, NX02-0020, and NX02-0022

	FT 2.5 mg		Placebo		*p* value
	*N*	%	*N*	%	
Patients with prostate cancer	349^a^	1.1 (4/349)	95^b^	5.3% (5/95)	0.0116^c^
Incidence of AUR	277^d^	1.08% (3/277)	142^e^	5.63% (8/142)	0.0058^c^

^a^Patients who received FT at least once in 0017, 0018, 0020, or 0022 with ≥ 4 years LF

^b^Patients who received Placebo in 0017/0018 and not CO to FT, with ≥ 4 years LF

^c^Chi-square test

^d^All patients in CO studies with ≥ 3 years follow-up

^e^Placebo patients in studies 0017/0018 not CO to FT, with ≥ 3 years follow-up

### Efficacy: primary endpoints

0017/0018 LF PO was met in both 0017/0018 and in the pooled studies (Table 2) (0017: FT mean − 5.1, median − 4.0 vs placebo − 3.8, − 3.5, *p* = 0.0378 (Wilcoxon); 0018: FT mean − 6.4, median − 6.3 vs placebo − 4.2, − 3, *p* < 0.0001 (Wilcoxon); pooled 0017/0018 FT mean − 5.7, median − 5.2 vs placebo − 4.0, − 3.0, *p* < 0.0001 (Wilcoxon). At 1 year there was significant FT group change from baseline but not versus placebo. *PO subgroup LF analyses* were statistically significant in all but one (BPH history ≥ 10 years) of adequately powered pre-planned subgroups. Except where noted, all of the following FT-treated subgroups showed statistically significant LF reductions in IPSS: Age; Ethnicity; Prior use of BPH treatment (Online Table 5); Prior medical therapy; BPH history; Baseline IPSS; Baseline PV; Baseline Qmax. Similar to the PO at 1 year, secondary endpoints showed significant differences from baseline in the FT groups but did not meet the 12 month secondary efficacy endpoints versus placebo. At 10 days post-treatment there was statistically significant difference in IPSS change from baseline versus placebo in 0017 (*p* = 0.0011) and in the pooled studies (*p* = 0.0032). *Additional Pre*-*Planned Efficacy Endpoints* (*online pages 2, 3, 9*): LF responder analysis was statistically significant (IPSS change < 0: 0017, *p* = 0.0099; 0018, *p* = 0.0023 pooled studies *p* < 0.0001. Responder analysis with cut-offs < − 1, < − 2, and < − 3 IPSS improvement were all *p* = < 0.0001 in the pooled studies (Online Table 6). *Pre*-*specified CO efficacy endpoints* in 0017/0018 patients who received FT re-injection in CO studies 0020/0022 are listed in Table 3. The LF co-POs were both significant: 1. 3-year incidence of BPH surgery in all CO FT (8.08%) versus all BPH medications (27.85%) (*p* < 0.0001); and 2. 3-year incidence of BPH surgery or AUR in placebo CO FT (6.07%) versus placebo with medications (33.3%) (*p* < 0.0001). *Supplementary analysis of PO Subgroup data:* (Online Table 7).

**Table 2:** Change in BPH Symptom Score (IPSS) from baseline to long-term follow-up

Visit	Statistic	NX02-0017			NX02-0018			Pooled studies NX02-0017 and NX02-0018		
		FT 2.5 mg	Placebo	*p* value^b^	FT 2.5 mg	Placebo	*p* value^b^	FT 2.5 mg	Placebo	*p* value^b^
		*N* = 292	*N* = 196		*N* = 290	*N* = 195		*N* = 582	*N* = 391	
Baseline^a^	*n*	292	196	–	290	195	–	582	391	–
	Mean (SD)	23.3(4.93)	23.1(5.04)	–	23.7 (4.98)	23.2 (5.13)	–	23.5 (4.96)	23.2 (5.08)	–
	Median	23.0	23.0	–	24.0	23.0	–	23.5	23.0	–
	Min–max	12.0–35.0	15.0–35.0	–	15.0 to 35.0	15.0 to 35.0	–	12.0 to 35.0	15.0 to 35.0	–
Change from Baseline (Visit 10)	*n*	292	196	–	290	195	–	582	391	–
	Mean (SD)	− 5.3(6.81)	− 6.1(7.35)	–	− 6.6 (6.41)	− 6.3 (6.83)	–	− 6.0 (6.64)	− 6.2 (7.09)	–
	Median	− 5.0	− 5.2	0.1871	− 5.0	− 6.0	0.5402	− 5.0	− 5.8	0.5760
	Min–max	− 27–9.0	− 29–14.0	–	− 26 to 10.0	− 28 to 9.0	–	− 27 to 10.0	− 29 to 14.0	–
Change from Baseline (Follow-up)	*n*	292	196	–	290	195	–	582	391	–
	Mean (SD)	− 5.1(6.23)	− 3.8(5.98)	–	− 6.4 (5.78)	− 4.2 (6.09)	–	− 5.7 (6.05)	− 4.0 (6.03)	–
	Median	− 4.0	− 3.5	0.0378	− 6.3	− 3.0	< 0.0001	− 5.2	− 3.0	< 0.0001
	Min–max	− 33–6.0	− 25 to 16.0	–	− 25 to 8.0	− 27 to 13.0	–	− 33 to 8.0	− 27 to 16.0	–

*N* number of patients within the population and treatment group based on the available observations

*n* number of available observations

^a^Baseline = Last available value before treatment

^b^Normality assumption not met and *p* value was based on non-parametric Wilcoxon rank sum test

**Table 3:** Crossover (CO) efficacy endpoints, studies NX02-0020 and NX02-0022

Endpoint	Groups	*p* value	Endpoint	Groups	*p* value
Surgery < 3 year	All BPH Meds (27.85%) versus FT CO (8.08%)^a^	< 0.0001	Surgery < 3 year	Placebo with BPH Meds (30.30%) versus Placebo FT CO (5.22%)	< 0.0001
Surgery or AUR < 3 year	Placebo with BPH Meds (33.33%) versus Placebo FT CO (6.09%)^b^	< 0.0001	Surgery < 3 year	Placebo not in CO (16.44%) versus FT CO (8.08%)	0.0100
Surgery < 2 year	Placebo with BPH Meds (17.14%) versus Placebo FT CO (0.78%)	< 0.0001	Surgery < 2 year	All BPH Meds (14.10%) versus FT CO(1.76%)	< 0.001
Surgery < 2 year	Placebo not in CO (9.04%) versus FT CO (1.76%)	0.0002	Surgery or AUR < 3 year	All BPH Meds (28.75%) versus FT CO (8.85%)	< 0.0001
Surgery or AUR < 3 year	Placebo not in CO (17.81%) versus FT CO (8.85%)	0.0078	Surgery or AUR < 2 year	Placebo with BPH Meds (20.00%) versus Placebo FT CO (1.56%)	< 0.0001
Surgery or AUR < 2 year	All BPH Meds (15.58%) versus FT CO(2.11%)	< 0.0001	Surgery or AUR < 2 year	Placebo not in CO (10.64%) versus FT CO (2.11%)	< 0.0001
IPSS Last Capture Responder	Placebo not in CO (48.68%) versus FT CO at last visit (80.70%)	< 0.0001	IPSS Last Capture Responder	Placebo not in CO (48.68%) versus Placebo FT CO at last visit (80.15%)	< 0.0001
IPSS Last Capture Responder	Placebo not in CO (48.68%) versus Last Data capture from 0020/0022, FU042015, FU022016 (64.36%)	0.0008	IPSS Last Capture Responder	Placebo not in CO (48.68%) versus Placebo FT CO, FU042015, FU022016 (67.52%)	0.0013
Surgery < 3 year	Placebo not in CO (16.44%) versus FT CO (5.22%)	0.0048	Surgery < 2 year	Placebo not in CO (9.04%) versus Placebo FT CO (0.78%)	0.0019
Surgery or AUR < 3 year	Placebo not in CO (18.37%) versus FT CO (6.09%)	0.0033	Surgery or AUR < 2 year	Placebo not in CO (10.64%) versus Placebo FT CO (1.56%)	0.0019
Nocturia change from baseline > 0	Placebo not in CO (22.16%) versus FT CO at last visit (12.03%)	0.0024	Nocturia change from baseline > 0	Placebo not in CO (22.16%) versus FT CO at last visit (12.37%)	0.0117

Group	*N*	IPSS Change from Baseline Mean (SD) median	*p* value versus Group 1	Group	*N*	IPSS Change from Baseline Mean (SD) median	*p* value versus Group 6
Group 1: Placebo patients not in CO	189	− 4.08 (7.73) 0.00		Group 6: Placebo patients not in CO with use of BPH medications	62	− 2.79 (5.92) − 1.00	
Group 2: All FT CO at last visit	316	− 8.02 (7.67) − 8.00	< 0.0001	Group 7: All CO patients at last visit	316	− 8.02 (7.67) − 8.00	< 0.0001
Group 3: Placebo FT CO at last visit	131	− 7.60 (7.41) − 6.00	< 0.0001	Group 8: Placebo patients in CO at last visit	131	− 7.60 (7.41) − 6.00	< 0.0001
Group 4: All FT CO patients last data capture	275	− 6.49 (8.15) − 5.00	0.0011	Group 9: All CO patients last visit capture	275	− 6.49 (8.15) − 5.00	0.0034
Group 5: Placebo CO patients last data capture	117	− 6.50 (7.90) − 6.00	0.0056	Group 10: Placebo CO patients last data capture	117	− 6.50 (7.90) − 6.00	0.0058

All other endpoints in Table 3 are secondary endpoints

*FU* follow-up, *CO* crossover BPH medications: conventional oral BPH medications 0017/0018: NX02-0017 and NX02-0018 studies

^a,b^Co-Primary Endpoints 1 and 2

## Discussion

The main findings of the 4 studies reported are summarized as follows: 1. LF symptomatic improvement from a single FT treatment under double blind conditions was statistically significant (mean IPSS − 5.7 points, placebo − 4.0, median − 5.2 vs − 3.0, *p* < 0.0001); 2. LF (3 year) FT reduction in incidence of BPH surgery in all CO patients (CO FT 8.08%) versus all non-CO patients with conventional oral medications was obtained (27.85%, p < 0.0001) (LT CO Primary Endpoint #1) (Table 3); 3. LF CO (3 year) reduction in incidence of spontaneous AUR (CO FT 1.08%, non-CO placebo 5.63%, *p* = 0.0058) was observed; 4. LF (4 year) reduction in incidence of PCa newly diagnosed after 12 months or later (FT 1.1%, placebo 5.3%, *p* = 0.0116) was observed; 5. LF IPSS improvement after single injection FT with later conventional oral medications (mean − 8.28 points) versus placebo with oral medications (− 4.74, *p* = 0.0094) was found; 6. LF IPSS improvement in first-line (treatment-naïve) patients (FT mean − 6.6 points, placebo − 4.0, median − 6.2 versus − 3.0, *p* < 0.0001) and in prior BPH treatment patients was found; 7. Sexual function improvements in first-line FT treated patients (+ 0.64 points) versus worsening (− 0.88 points) in placebo patients (*p* = 0.0049); 8. LF (3 year) reduction in the incidence of BPH surgery or AUR (placebo CO FT 6.09% versus placebo CO conventional medications 33.3%, *p* < 0.0001) (LF CO Primary Endpoint #2) (Table 3); 9. LF superior FT responder results at all four IPSS improvement cut-offs − 1 to − 4 vs placebo (*p* < 0.0001). Although these studies were designed primarily as placebo controlled prospective randomized double blind parallel group trials and not as head-to-head comparator studies versus oral BPH treatments, there was extensive LT protocol follow-up which included in part comparator analysis of FT versus standard oral therapies in relation to outcomes of long-term IPSS change, incidence of BPH surgery, and AUR.

On the basis of 351 patients from studies 0017/0018, with patient individual discretion to return for elective re-injection of FT in studies 0020/0022, the administration of FT during a routine TRUS was considered well tolerated. Earlier trials [17, 18] feedback and questionnaires (NX02-0016, clinicaltrials.gov NCT00759135) had indicated the injection was not considered painful by patients (unpublished data).

Safety findings thus far indicate no known molecular side effects from FT. AE data and laboratory results were similar in FT and placebo groups. The only ‎reported related AEs in both drug and placebo groups were from prophylactic antibiotic treatments and from the transrectal needle insertion; in both of these categories the events reported were mainly mild, transient and of infrequent requirement of additional treatment. There were no cases of urosepsis in FT-treated patients, and the rate of UTI was not increased compared to placebo. There were no significant sexual side effects reported, and semen analysis showed no significant changes from baseline. Anti-FT antibodies were undetectable. PK analysis showed no extraprostatic signal at any time point post-injection, indicating that FT has no measurable contact with tissues outside prostate.

These studies demonstrate statistically significant objective and subjective efficacy data. The PO variable (IPSS), responder rates, sexual function, and the prospective IPSS subgroups data are more subjective, while other efficacy variables (incidence outcomes of surgical treatment for BPH, AUR, prostate cancer) are objective. LF IPSS improvement was statistically significant in all studies, IPSS LF responder rates were statistically significant for all improvement cut-offs, and sexual function LF in placebo patients was mean worsening (− 0.88) compared to mean improvement in FT treated first line prior BPH treatment naïve patients (+ 0.64, *p* = 0.004). Several important FT pre-specified IPSS sub-groups showed significant LF results superior to placebo, including treatment naïve first-line patients (median − 6.2 vs placebo − 3.0, *p* < 0.0001). Pre-specified objective outcome analyses yielded consistently positive results in terms of reduced LF incidence of BPH surgery, reduced incidence of spontaneous AUR, and reduction in prostate cancer diagnosis. The LF FT re-injection studies also demonstrated statistically significant improvements compared to placebo groups in all of multiple endpoints of IPSS; in responder analysis; and in nocturia frequency improvement. Both the 2 pre-specified Primary Endpoints and all the 28 pre-specified Secondary Endpoints in the LF CO analysis were statistically significant, showing clearly better outcomes after 24 and 36 months for all patient groups who received an injection of FT in Studies 0020/0022, as compared to groups of all non-CO placebo, or as compared to placebo who crossed over to conventional oral medications.

The published literature indicates that 1-year outcomes in BPH studies are less valid than previously assumed, insofar as strong placebo responses and effects are constantly encountered up to 1 year and perhaps longer [19–22]. The literature further indicates that long-term (> 1 year) data capture and analyses are required to validate BPH treatments, given also that BPH is a condition that requires chronic treatment [19]. The results reported here are consistent with this well-described effect. Although there were statistical differences from baseline in FT-treated patients in all endpoints, there were no statistical differences from placebo at 1 year. The reasons for the first year strong placebo effect have been postulated to include regression to the mean and other hypotheses (patient and physician expectations, activation of reward circuitry, release of endogenous opioids, dopamine and other transmitters, psychological conditioning) [19–22]. In the studies reported herein, placebo Qmax improvement from baseline was 1.9 mL/s which is similar in range to oral BPH medications, and if Qmax is considered a non-subjective outcome, it further suggests that placebo is probably at least partially active. Placebo injections into the prostate have been considered to be active controls in the reported literature due to a “mass effect” or to a change in prostate parenchyma or capsule, which may also in part explain the pronounced effect up to 1 year in all published trials of this nature [22].

Reduction in PCa incidence rate in FT-treated BPH patients was statistically significant. Patients in CO FT 0020/0022 had incidence after 4 years of 1.1% compared to non-CO placebo incidence of 5.3% (*p* = 0.0116). FT 2.5 and 15 mg single targeted injections to biopsy proven prostate cancer foci have been found to reduce clinical and biopsy progression and treatment outcome parameters in T1c prostate cancer patients in a multi-year study of 146 patients plus crossovers (Nymox, clinicaltrial.gov NCT # NCT01620515, data on file). Although the present BPH studies used TRUS targeted to the transition zone, it is possible that the 10 mL injections also entered to some extent the peripheral prostate zones where PCa is usually found. Significant PCa reduction in the BPH studies reported here suggests that FT may have an inhibitory effect on clinically undetected low-grade PCa microfoci and/or precursor cells and lesions in quadrant locations where the FT BPH injections were bioavailable, but further study will be required to clarify the mechanism(s) of action and corroborate this finding.

Although these FT studies are the largest and longest duration clinical trials to have been completed for an intraprostatic injectable for BPH, there are limitations to the number of adequately powered prospective subgroups that are possible. The efficacy and safety of FT combination treatments, of FT used in different patient study populations, the variability of dosing schedule remain to be answered by additional investigations. The inclusion criteria precluded enrollment of patients with more severe advanced BPH. It will be important, for example, to test FT in subjects who are in chronic retention due to BPH. Patients who have intractable severe LUTS but are poor surgical candidates are another important group where investigation may be warranted. Further studies will be needed to determine the impact of FT in relation to the gold standard TURP.

In conclusion, data from these 4 U.S. studies show statistically significant long-term improvement in BPH symptoms and objective outcomes including significant reduction in spontaneous AUR as well as the addition of BPH surgery; thus far (after a total of > 1700 patient treatments including FT and placebo in U.S. trials to date since 2002) FT is well tolerated with an excellent safety profile. Hence, FT is a safe and efficacious clinic-based treatment for BPH involving an intraprostatic injection that requires only a few minutes to administer, with no catheter nor anesthesia requirements. FT injectable represents a novel, first in class BPH treatment modality.

## Electronic supplementary material

Below is the link to the electronic supplementary material.

Supplementary material 1 (DOCX 3763 kb)
