# Supplementary material for: Fexapotide triflutate: results of long-term safety and efficacy trials of a novel injectable therapy for symptomatic prostate enlargement
Source: World J Urol. 2018 Jan 29;36(5):801–9. doi: 10.1007/s00345-018-2185-y (PMC5916987; doi:10.1007/s00345-018-2185-y)

**Online Additional Pages**

**Fexapotide Triflutate: Results of Long-Term Safety and Efficacy Trials of a Novel Injectable Therapy for Symptomatic Prostate Enlargement**

Neal Shore^1*^, Ronald Tutrone^2^, Mitchell Efros^3^, Mohamed Bidair^4^, Barton Wachs^5^, Susan Kalota^6^, Sheldon Freedman^7^, James Bailen^8^, Richard Levin^9^, Stephen Richardson^10^, Jed Kaminetsky^11^, Jeffrey Snyder^12^, Barry Shepard^13^, Kenneth Goldberg^14^, Alan Hay^15^, Steven Gange^16^, Ivan Grunberger^17^

^1^Carolina Urologic Research Center, Myrtle Beach, SC; ^2^Chesapeake Urology, Baltimore, MD; ^3^Accumed Research, Garden City, NY; ^4^San Diego Clinical Trials, San Diego, CA; ^5^Atlantic Urology Medical Group, Long Beach, CA; ^6^Urological Associates of Southern Arizona, Tucson, AZ; ^7^Freedman Urology, Las Vegas, NV; ^8^First Urology, Louisville, KY; ^9^Chesapeake Urology, Towson, MD; ^10^Jean Brown Research, Salt Lake City, UT; ^11^University Urology, New York, NY; ^12^Genitourinary Surgical Consultants, Denver, CO; ^13^Urological Surgeons of Long Island, Garden City, NY; ^14^U T Southwestern Dept of Urology, Lewisville, TX; ^15^Willamette Urology, Salem, OR; ^16^Summit Urology Group, Salt Lake City, UT; ^17^Brooklyn Urology, Brooklyn, NY

*Corresponding author (NShore@gsuro.com)

Funded by Nymox Pharmaceutical Corporation

**Online Additional to Patients and Methods:**

*Laboratory analyses:* Plasma samples for pharmacokinetic (PK)‎ assays were drawn from patients in studies 0020 and 0022, at 1, 5, 10, and 20 minutes post-injection (n = 106), immediately stored frozen at ≤-20^0^C and transported to a central laboratory for measurement (Pacific Biolabs, Hercules, CA). Semen samples for analysis were provided by consecutive volunteers in Studies 0020 and 0022 (n=41 samples from FT treated patients; n=32 placebo), and tested at local institution laboratories (volume, sperm count, motility, maturation). Plasma samples for anti-FT antibodies from patients in Studies 0017, 0018, 0020 and 0022 were drawn pre- and post-treatment (1 year) (n=1254 samples) and tested at a central laboratory (Southern Research Institute, Birmingham AL). A blinded qualified central reader was used to interpret all Qmax readings (A. Singla, University of Toledo, Toledo, OH). A central laboratory was used for all hematology and clinical chemistry analyses (LabCorp Clinical Trials, Cranford, NJ).  Urine and blood samples were also analyzed locally in the first 2 weeks post-treatment as a precaution in order to expedite treatment based on any potentially positive results. Pre-randomization rectal stool swabs for culture for antibiotic sensitivities were done at UC Medical Center Department of Pathology & Laboratory Medicine, Orange, CA.

Descriptive statistics for the primary efficacy variable were to be tabulated by treatment group. The comparison between treatment groups was assessed using a t-test if data was normally distributed; otherwise a non-parametric test was applied. Superiority of FT to placebo was established if the p-value was less than or equal to 0.05. The changes from baseline to 90 days (Secondary endpoint) (Visit 7), 180 days (Visit 8), and 270 days (Visit 9) in IPSS were also assessed. Qmax (Secondary endpoint at 90 days and 12 months) in the first 12 months was to be analyzed by study visit and treatment group. All available non-reinjected patients with ≥2 years post-FT treatment interval were eligible for follow-up assessment. Long-term follow-up prospective pre-planned safety analysis included additional analysis of adverse events of pre-specified special interest relevant to efficacy conclusions: 1. Long-term incidence of prostate cancer; 2. Long-term incidence of spontaneous acute urinary retention; 3. Incidence of sexual side effects. Additional efficacy endpoints were to be evaluated in each study, including responder analysis for IPSS, changes from baseline in PV, BII, SFQ, quality of life question (QOL), time-weighted change in IPSS. The responder analysis for IPSS included IPSS improvement (changes <0, <-1, <- 2, <-3), from baseline to 12 months and long-term follow-up (2-6.75 years, mean 3.58 years, median 3.67 years; FT re-injection CO mean 4.27 years, median 4.42 years). The post-randomization incidences were listed and summarized descriptively by treatment group for the individual studies and for the pooled studies. Differences in the above incidences between the treatment groups were assessed using chi-square test. All other additional efficacy endpoints were presented by treatment group for the individual studies and for the pooled study. Differences between the treatment groups were assessed using t-test if data was normally distributed; otherwise a non-parametric test was applied. The primary endpoint subgroup analyses were further conducted for the individual and pooled datasets (ITT population) according to age; race; ethnicity; prior use of BPH therapy (treatment naïve; prior conventional medical therapy); BPH history (< 10 years; ≥ 10 years); baseline disease severity (IPSS Score <20; ≥20); baseline prostate volume (<40; ≥40); Qmax (<5; ≥5). Statistical analysis was performed by Amarex Clinical Research LLP (Germantown, MD). Database for 0017 and 0018 was designed by PharPoint Research Inc (Durham, NC).

**Online Additional to Results**

BII: In both 0017 and 0018 there was statistically significant difference in the change in mean BII from baseline to Visit 5 at 10 days (0017, p=.0197; 0018, p=.0302; pooled p=.0015) and in mean BII from baseline to 90 days in the pooled studies (p =.0498). BII was not assessed long-term. Prostate Volume: In study 0017 prostate volume showed a statistically significant reduction from baseline in the FT treated group at 12 months (-3.46%) (p<0.0001). Pooled studies showed significant prostate volume reduction from baseline in FT treated patients (-2.06%) (p=0.0003). Placebo groups in the pooled studies showed no significant difference from baseline (-0.9%) (p=0.1262). PV was not assessed long-term. SFQ: The treatment-naïve subgroup in 0017 and 0018 showed significant mean improvement from baseline in the FT treated group (0017 p=.0436, 0018 p=.0005, pooled studies p<.0001). At long-term follow-up, treatment-naive patients treated with FT showed statistically significant improvement from baseline (+0.64 points p=.0449) compared to the placebo where there was worsening (-0.88, p=.0434). The difference between FT and placebo at long-term follow-up in the treatment-naïve patients was statistically significant (p=.0049) (Online Table 8).

**Online Additional Tables**

Online Table 1. Inclusion/Exclusion Criteria for Studies NX02-0017 and NX02-0018

Online Table 2. Baseline Characteristics, NX02-0017 and NX02-0018 Studies, ITT Population

Online Table 3. Demographic Characteristics, NX02-0017 and NX02-0018 Studies, ITT Population

Online Table 4. Patient Disposition, NX02-0017 and NX02-0018 Studies, All Patients at 12 Months

Online Table 5. Change in IPSS in Treatment-Naïve and Prior Treatment Subgroups

Online Table 6. Responder Analysis for IPSS, Pooled Studies, ITT Population

Online Table 7. Change in Self-Reported Sexual Function (After Mean 21 Months)

Online Table 8. Change in IPSS in Subgroup of Patients Receiving Subsequent Oral BPH Medications, and in Subgroup of Patients Receiving Subsequent Oral BPH Medications or BPH Surgery

Online Table 9. Clinical Trial Sites

**Online Additional Figure Legends**

Online Figure 1. Crossover Re-injection Studies Flowchart

Online Figure 2. A. Normal rat prostate gland, Hematoxylin-eosin, X 400

B. Rat injected with FT 1 mg/mL, showing extensive apoptotic cell loss after 24 hours. Hematoxylin-eosin, X400

C. Rat prostate after FT single injection 1 mg/mL, showing near total loss of prostatic glandular epithelial cell population and marked shrinkage of gland. Hematoxylin-eosin, X100

D-F. Rat prostate 12 months after FT single injection 1 mg/mL, showing normal surviving nerve fibers (arrows) in fields with total loss of prostate glandular epithelium. Hematoxylin-eosin, X400

(Figure courtesy of Nymox Corp.)

Online Figure 3. A, B. LNCAP prostate cancer cells in vitro 24 hours post treatment with FT 2.5 mg/mL, labeled with bound Annexin V-biotin, viewed under UV light, X600. Green fluorescence indicates cells undergoing apoptosis

C. Prostate biopsy from patient with T1c prostate cancer 6 weeks after FT injection 10 mL, 1.5 mg/mL into tumor. TUNEL stain, X400.  Dark brown foci are TUNEL positive immunoperoxidase staining indicating cellular apoptosis

D. Apoptotic cell in vitro showing deeply convoluted nuclear invaginations and grooving (arrows). LNCAP prostate cancer cell line in vitro 24 hours post treatment with FT 2.5 mg/mL. Electron microscopy, X12,000

E. Massive apoptotic nuclear bleb (NB) in LNCAP prostate cancer cell line in vitro 24 hours post treatment with FT 2.5 mg/mL. Electron microscopy, X20,000. N nucleus

(Figure courtesy of Nymox Corp.)

**Online Additional Table 1. Inclusion/Exclusion Criteria for Studies NX02-0017 and NX02-0018**

| **Inclusion Criteria**  Male, ≥45 years of age, with signed informed consent.  No clinically significant deviation from normal in medical history, physical examination, clinical laboratory determinations and ECG.  History of BPH ≥1 year; AUASI ≥15; PV ≥30 mL (30 g) and ≤ 70 mL (70 g) as determined by TRUS ≤ 6 mos prior, Qmax < 15 mL/s.  No BPH medications prior to baseline assessments (AB ≥2 weeks, and 5-ARI ≥ 6 mos stopped prior), and for trial duration. |
| --- |
| **Exclusion Criteria**  Acute or chronic prostatitis or suspected prostatitis.  History or evidence from physical examination, clinical laboratory tests, or electrocardiogram of any acute or chronic disease that may interfere with the study or endanger the patient, or that would interfere with the patient’s ability to provide informed consent, to comply with study instructions, provide an objective assessment of his symptoms, or that might confound the interpretation of the study results. Any other sound medical, psychiatric and/or social reason (including desire to maintain fertility) as determined by the investigator. History of any acute illness in the 15 days preceding screening. Participation in a study of any investigational drug or device within the previous 90 days.  Use of any of the following concomitant medications: immunosuppressants, anticoagulants, alpha-blockers, 5 alpha-reductase inhibitors, anti-psychotics, chemotherapy, medication prescribed for dementia, male hormonal replacement, and medication prescribed for overactive bladder. Use of any new prescription or over-the-counter medications and herbal preparations within 1 week prior to Visit 2.  History of any significant drug allergy.  Documented urinary tract infection more than once in the past 12 months.  PSA ≥ 10 ng/mL. For patients with PSA ≥4 ng/mL and <10 ng/mL a negative prostatic biopsy within prior 12 months.  Presence of a symptomatic median lobe of the prostate.  Any urethral disease or condition. Prostate or bladder cancer. History of pelvic irradiation. History of neurogenic bladder or LUTS secondary to neurologic disease. History of central nervous system injuries (including stroke or spinal cord injury) within 6 months of Visit 1. History of urinary retention in the previous 12 months. History of use of self-catheterization for urinary retention. Clinical evidence of Mullerian duct cysts or atonic, decompensated, or hypocontractile bladder.  Previous surgical or invasive prostate treatments such as TURP, TUMT, TUNA, laser or any other minimally invasive treatment. History of pelvic trauma or surgery of any type including bowel resection. Clinically significant microscopic hematuria that has not been evaluated by a urologist and has not been attributed to BPH.  Post-void residual urine volume >200 mL. Lower urinary tract instrumentation of any type within 30 days of Visit 1.  Clinically significant renal or hepatic impairment; bleeding disorder; uncontrolled diabetes type 1 or type 2; history of drug, alcohol, or other substance abuse within 6 months of Visit 1; any immunosuppressive disorders or conditions. |

| **Online Additional Table 2. Baseline Characteristics, NX02-0017 and NX02-0018 Studies, ITT Population** | | | | | | |
| --- | --- | --- | --- | --- | --- | --- |
| **Parameter** |  | **NX02-0017** | |  | **NX02-0018** | |
|  |  | **FT 2.5 mg** | **Placebo** |  | **FT 2.5 mg** | **Placebo** |
|  |  | **N=292** | **N=196** |  | **N=290** | **N=195** |
|  | **Characteristic** | **n (%)** | **n (%)** |  | **n (%)** | **n (%)** |
| **Prior BPH Treatment** | Previous BPH Treatment | 176 (60.3) | 116 (59.2) |  | 179 (61.7) | 110 (56.4) |
|  | Treatment Naïve | 116 (39.7) | 80 (40.8) |  | 111 (38.3) | 85 (43.6) |
| **BPH History** (number of years between onset and randomization) | < 10 years | 226 (77.4) | 157 (80.1) |  | 214 (73.8) | 164 (84.1) |
|  | ≥ 10 years | 66 (22.6) | 38 (19.4) |  | 70 (24.1) | 28 (14.4) |
|  | Missing | 0 ( 0.0) | 1 ( 0.5) |  | 6 ( 2.1) | 3 ( 1.5) |
| **Baseline Disease Severity**  (Baseline BPH Symptom Score) | < 20 | 68 (23.3) | 50 (25.5) |  | 73 (25.2) | 55 (28.2) |
|  | ≥ 20 | 224 (76.7) | 146 (74.5) |  | 217 (74.8) | 140 (71.8) |
| **Baseline Prostate Volume (g)** | < 40 | 147 (50.3) | 100 (51.0) |  | 134 (46.2) | 89 (45.6) |
|  | ≥ 40 | 145 (49.7) | 96 (49.0) |  | 156 (53.8) | 105 (53.8) |
|  | Missing | 0 ( 0.0) | 0 ( 0.0) |  | 0 ( 0.0) | 1 ( 0.5) |
| **Baseline Urinary Peak Flow Rate (mL/sec) [1]** | < 5 | 12 ( 4.1) | 9 ( 4.6) |  | 7 ( 2.4) | 4 ( 2.1) |
|  | ≥ 5 | 278 (95.2) | 187 (95.4) |  | 282 (97.2) | 190 (97.4) |
|  | Missing | 2 ( 0.7) | 0 ( 0.0) |  | 1 ( 0.3) | 1 ( 0.5) |
| **Baseline Urinary Peak Flow Rate (mL/sec) [2]** | < 5 | 15 ( 5.1) | 8 ( 4.1) |  | 7 ( 2.4) | 4 ( 2.1) |
|  | ≥ 5 | 275 (94.2) | 188 (95.9) |  | 282 (97.2) | 190 (97.4) |
|  | Missing | 2 ( 0.7) | 0 ( 0.0) |  | 1 ( 0.3) | 1 ( 0.5) |
| N=number of patients within the ITT population; the denominator for percentages. | | | | | | |
| n = number of observed patients; the numerator for percentages. | | | | | | |
| [1] Baseline 1 = Second peak flow 3 days before treatment. | | | | | | |
| [2] Baseline 2 = Mean of the 2 pre-treatment qualifying central reader measurements. | | | | | | |

| **Online Additional Table 3. Demographic Characteristics, NX02-0017 and NX02-0018 Studies, ITT Population** | | | | | | |
| --- | --- | --- | --- | --- | --- | --- |
|  |  | **NX02-0017** | |  | **NX02-0018** | |
|  |  | **Controlled** | |  | **Controlled** | |
|  |  | **FT 2.5 mg** | **Placebo** |  | **FT 2.5 mg** | **Placebo** |
| **Parameter** | **Characteristic** | **N=292** | **N=196** |  | **N=290** | **N=195** |
| **Race, n (%)** | White | 262 (89.7) | 176 (89.8) |  | 251 (86.6) | 174 (89.2) |
|  | Black or African American | 23 ( 7.9) | 15 ( 7.7) |  | 25 ( 8.6) | 16 ( 8.2) |
|  | Other | 4 ( 1.4) | 2 ( 1.0) |  | 6 ( 2.1) | 2 ( 1.0) |
|  | Asian | 3 ( 1.0) | 2 ( 1.0) |  | 5 ( 1.7) | 1 ( 0.5) |
|  | American Indian or Alaska Native | 0 ( 0.0) | 1 ( 0.5) |  | 1 ( 0.3) | 2 ( 1.0) |
|  | Native Hawaiian or Other Pacific Islander | 0 ( 0.0) | 0 ( 0.0) |  | 1 ( 0.3) | 0 ( 0.0) |
|  | Missing | 0 ( 0.0) | 0 ( 0.0) |  | 1 ( 0.3) | 0 ( 0.0) |
| **Ethnicity, n (%)** | Not Hispanic or Latino | 277 (94.9) | 184 (93.9) |  | 270 (93.1) | 185 (94.9) |
|  | Hispanic or Latino | 15 ( 5.1) | 12 ( 6.1) |  | 20 ( 6.9) | 10 ( 5.1) |
|  | Missing | 0 ( 0.0) | 0 ( 0.0) |  | 0 ( 0.0) | 0 ( 0.0) |
| **Age (years)** | n | 292 | 196 |  | 290 | 195 |
|  | Mean (SD) | 62.1 ( 8.2) | 63.5 ( 8.2) |  | 62.5 ( 8.7) | 62.5 ( 7.8) |
|  | Median | 62.0 | 63.0 |  | 62.0 | 62.0 |
|  | Min - Max | 45.0-83.0 | 46.0-87.0 |  | 45.0-84.0 | 45.0-82.0 |
| **Age (years) - by Decade, n (%)** | ≥40 to <50 | 15 ( 5.1) | 8 ( 4.1) |  | 18 ( 6.2) | 7 ( 3.6) |
|  | ≥50 to <60 | 97 (33.2) | 56 (28.6) |  | 98 (33.8) | 64 (32.8) |
|  | ≥60 to <70 | 124 (42.5) | 87 (44.4) |  | 108 (37.2) | 89 (45.6) |
|  | ≥70 | 56 (19.2) | 45 (23.0) |  | 66 (22.8) | 35 (17.9) |
| N=number of patients within the population and treatment group; the denominator for percentages. | | | | | | |
| n = number of observed patients; the numerator for percentages. | | | | | | |

**Online Additional Table 4. Patient Disposition, NX02-0017 and NX02-0018 Studies, All Patients at 12 Months**

| **Parameter** | **NX02-0017** | |  | **NX02-0018** | |
| --- | --- | --- | --- | --- | --- |
|  | **FT 2.5 mg** | **Placebo** |  | **FT 2.5 mg** | **Placebo** |
|  | **N=299** | **N=199** |  | **N=297** | **N=200** |
|  | **n (%)** | **n (%)** |  | **n (%)** | **n (%)** |
| **Randomized** | 299 | 199 |  | 297 | 200 |
| **Received Treatment** | 294 (98.3) | 196 (98.5) |  | 292 (98.3) | 195 (97.5) |
| **Completed the Study** | 266 (89.0) | 183 (92.0) |  | 268 (90.2) | 174 (87.0) |
| **Reason for Premature Withdrawal/Discontinuation** | - | - |  | - | - |
| Withdrew Consent | 10 ( 3.3) | 6 ( 3.0) |  | 12 ( 4.0) | 13 ( 6.5) |
| Lost to Follow-up | 7 ( 2.3) | 2 ( 1.0) |  | 7 ( 2.4) | 3 ( 1.5) |
| Adverse Event | 3 ( 1.0) | 2 ( 1.0) |  | 2 ( 0.7) | 2 ( 1.0) |
| Other | 1 ( 0.3) | 0 ( 0.0) |  | 3 ( 1.0) | 2 ( 1.0) |
| Protocol Violation - Non compliance | 4 ( 1.3) | 2 ( 1.0) |  | 0 ( 0.0) | 1 ( 0.5) |
| Missing | 0 ( 0.0) | 1 ( 0.5) |  | 0 ( 0.0) | 0 ( 0.0) |
| No Longer Meets Criteria | 3 ( 1.0) | 0 ( 0.0) |  | 0 ( 0.0) | 0 ( 0.0) |
| N=number of patients randomized; the denominator for percentages. | | | | | |
| n = number of observed patients; the numerator for percentages. | | | | | |

**Online Additional Table 5. Change in IPSS in Treatment-Naïve and Prior Treatment Subgroups**

|  |  | **Treatment Naïve Subgroup** | | |  | **Prior Conventional Medical Therapy Subgroup** | | |
| --- | --- | --- | --- | --- | --- | --- | --- | --- |
|  |  | **FT 2.5 mg** | **Placebo** | **p-Value[2]** |  | **FT 2.5 mg** | **Placebo** | **p-Value[2]** |
| **Visit** | **Statistic** | **N=227** | **N=165** |  |  | **N=355** | **N=226** |  |
| **Baseline [1]** | n | 227 | 165 | - |  | 355 | 226 | - |
|  | Mean (SD) | 23.3(4.94) | 22.3(4.95) | - |  | 23.6(4.97) | 23.8(5.09) | - |
|  | Median | 23.0 | 22.0 | - |  | 24.0 | 24.0 | - |
|  | Min - Max | 12.0 - 34.0 | 15.0 - 35.0 | - |  | 15.0 - 35.0 | 15.0 - 35.0 | - |
| **Change from Baseline (Visit 10)** | n | 227 | 165 | - |  | 355 | 226 | - |
|  | Mean (SD) | -7.0(6.70) | -6.0(7.18) | - |  | -5.3(6.52) | -6.3(7.04) | - |
|  | Median | -6.0 | -6.0 | 0.2147 |  | -4.6 | -5.7 | 0.0840 |
|  | Min - Max | -27 - 10.0 | -28 - 14.0 | - |  | -25 - 9.0 | -29 - 9.0 | - |
| **Change from Baseline (Follow-up)** | n | 227 | 165 | - |  | 355 | 226 | - |
|  | Mean (SD) | -6.6(5.68) | -4.0(6.66) | - |  | -5.2(6.21) | -4.0(5.54) | - |
|  | Median | -6.2 | -3.0 | < 0.0001 |  | -4.0 | -3.2 | 0.0242 |
|  | Min - Max | -25 - 8.0 | -25 - 16.0 | - |  | -33 - 6.0 | -27 - 11.0 | - |
| N=number of patients within the population and treatment group based on the available observations. | | | | | | | | |
| n = number of available observations. | | | | | | | | |
| [1] Baseline = Last available value before treatment for all patients. | | | | | | | | |
| [2] Normality assumption not met and p-value was based on non-parametric Wilcoxon rank sum test. | | | | | | | | |
| SD = Standard Deviation; Min = Minimum Value; Max = Maximum Value. | | | | | | | | |

**Online Additional Table 6. Responder Analysis for AUASI, Pooled Studies, ITT Population**

|  |  | **FT 2.5 mg** | **Placebo** |
| --- | --- | --- | --- |
| **Visit** | **Statistic** | **N=582** | **N=391** |
| **Follow-Up** | Improvement (Change < 0) | 464(79.73%)^[1]^ | 268 (68.54%) |
|  | Worsening or Lack of Improvement (Change ≥ 0) | 118(20.27%) | 123 (31.46%) |
|  | Improvement (Change < -1) | 439(75.43%)^[1]^ | 250 (63.94%) |
|  | Worsening or Lack of Improvement (Change ≥ -1) | 143(24.57%) | 141 (36.03%) |
|  | Improvement (Change < -2) | 406(69.76%)^[1]^ | 218 (55.75%) |
|  | Worsening or Lack of Improvement (Change ≥ -2) | 176(30.24%) | 173 (44.25%) |
|  | Improvement (Change < -3) | 363(62.37%)^[1]^ | 194 (49.62%) |
|  | Worsening or Lack of Improvement (Change ≥ -3) | 219(37.63%) | 197 (50.38%) |
| N=number of patients within the population and treatment group based on the available observations. | | | |
| [1] p<.0001 chi-square test. | | | |

Online Additional Table 7. Change in Self-Reported Sexual Function (After Mean 21 Months)^[1]^

| **Sexual Function Measure** | **Treatment-Naive Patients Treated With fexapotide triflutate 2.5 mg (n=88)** | **Treatment-Naïve Patients Treated with Placebo**  **(n=68)** |
| --- | --- | --- |
| Change from Baseline in SFQ score (after mean 21 months) | +0.64 (2.93)^[2]^ | -0.88 (3.53) |

[1] All treatment naïve subjects in NX02-0017 and NX02-0018 with untreated long-term baseline sexual function questionnaire values in NX02-0020 and NX02-0022

[2] p=0.0049 t-test

**Online Additional Table 8. Change in IPSS in Subgroup of Patients Receiving Subsequent Oral BPH Medications, and in Subgroup of Patients Receiving Subsequent Oral BPH Medications or BPH Surgery**

|  |  | **0017 and 0018 Studies Pooled** | | | | | | |
| --- | --- | --- | --- | --- | --- | --- | --- | --- |
|  |  | **Patients Receiving Subsequent BPH Medications Subgroup** | | |  | **Patients Receiving Surgery or Subsequent BPH Medications Subgroup** | | |
|  |  | **FT 2.5 mg** | **Placebo** | **p-Value [2]** |  | **FT 2.5 mg** | **Placebo** | **p-Value [2]** |
| **Visit** | **Statistic** | **N=68** | **N=42** |  |  | **N=93** | **N=60** |  |
| **Baseline [1]** | n | 68 | 42 |  |  | 93 | 60 |  |
|  | Mean (SD) | 24.38(4.31) | 22.17(5.08) |  |  | 24.73(4.26) | 22.95(5.52) |  |
|  | Median | 24.0 | 22.0 |  |  | 24.0 | 22.0 |  |
|  | Min - Max | 15.0 - 34.0 | 15.0 - 34.0 |  |  | 15.0 - 34.0 | 15.0 - 34.0 |  |
| **Change from Baseline (Follow-up)** | n | 68 | 42 |  |  | 93 | 60 |  |
|  | Mean (SD) | -8.28(7.04) | -4.74(6.66) | 0.0094 |  | -10.6(8.35) | -7.40(8.96) | 0.0308 |
|  | Median | -8.0 | -5.0 |  |  | -10.0 | -6.5 |  |
|  | Min - Max | -27 - 5.0 | -16 - 16.0 |  |  | -31 - 5.0 | -33 - 16.0 |  |
| [1] Baseline = Last available value before treatment | | | | |  |  |  |  |
| [2] t-test | | | | |  |  |  |  |

**Online Additional Figure 1. Crossover Re-injection Studies Flowchart**

1

2

3

4

5

6

7

8

9

10

0017/0018 ACTIVE

n = 586

0020/0022 ACTIVE

n = 208

NO TREATMENT (DID NOT ENROLL IN 0020/0022)

n = 378

0017/0018 PLACEBO

n = 391

0020/0022 ACTIVE

n = 143

NO TREATMENT (DID NOT ENROLL IN 0020/0022)

n = 248

USED APPROVED DRUG^a^

n = 91

NO APPROVED DRUG USED^b^

n = 287

USED APPROVED DRUG^c^

n = 62

NO APPROVED DRUG USED^d^

n = 186

a. n=70 within first 3 years post-randomization in 0017/0018; n=83 within 4 years.

b. n=143 within first 3 years post-randomization in 0017/0018 with a minimum of 3 years follow-up; n=83 within 4 years with ≥4 years follow-up.

c. n=47 within first 3 years post-randomization in 0017/0018; n=54 within 4 years.

d. n=95 within first 3 years post-randomization in 0017/0018 with a minimum of 3 years follow-up; n=62 within 4 years with ≥4 years follow-up.

**Figure 2**


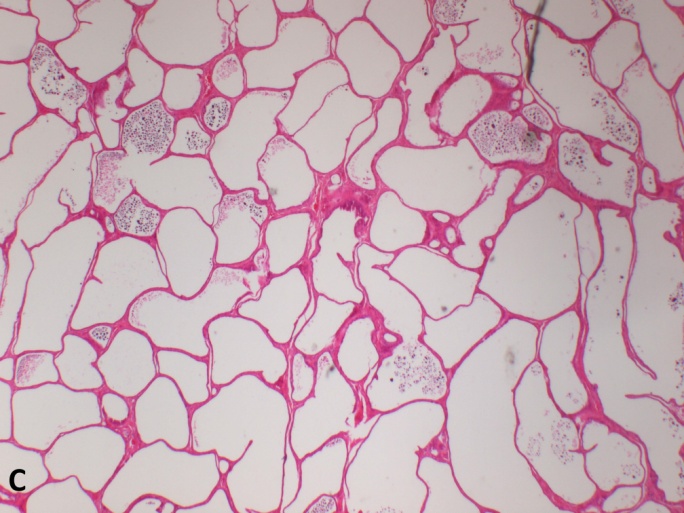

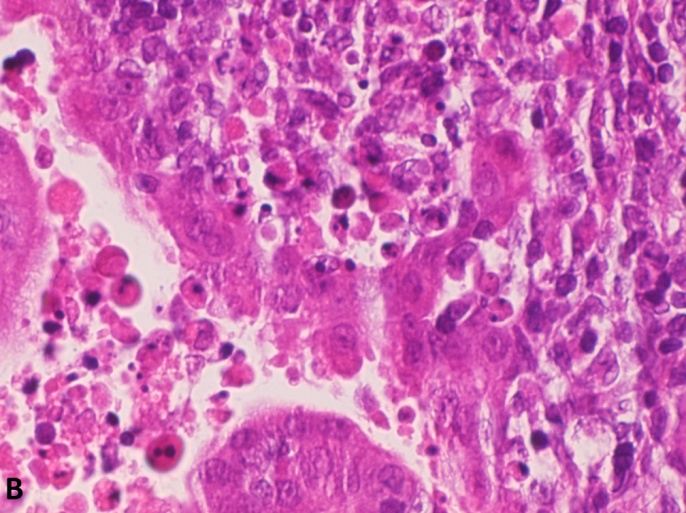

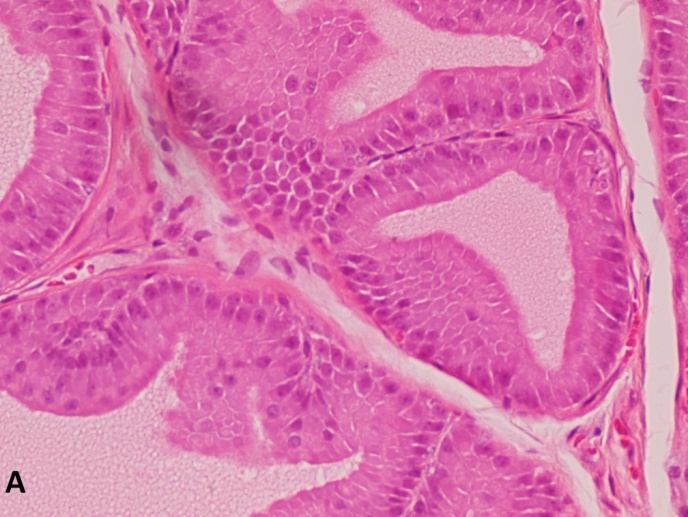


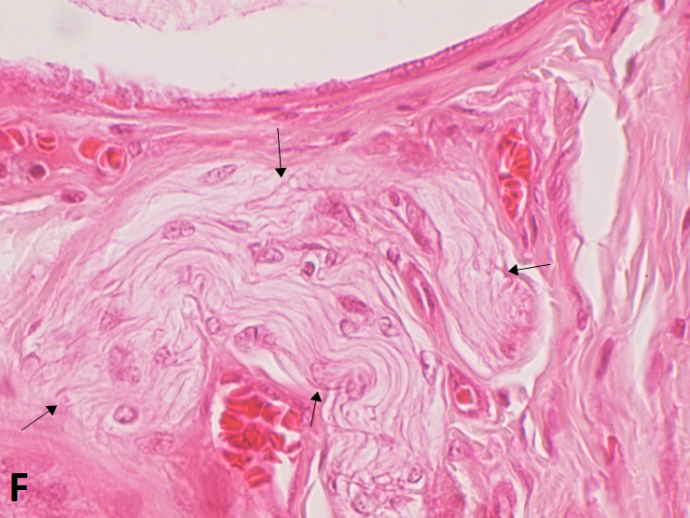

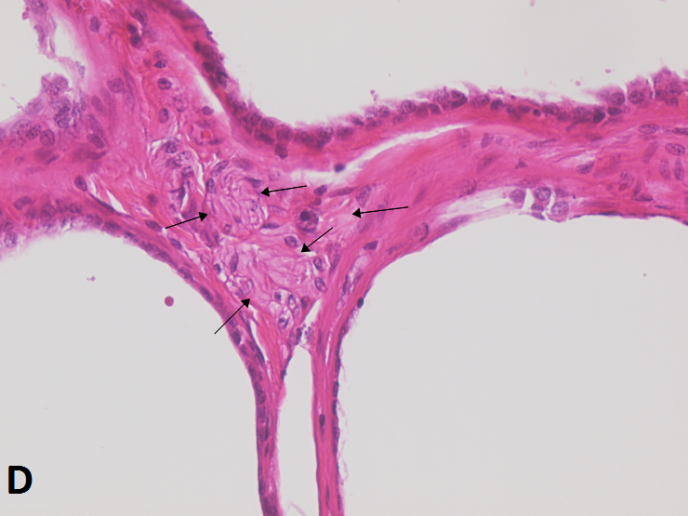

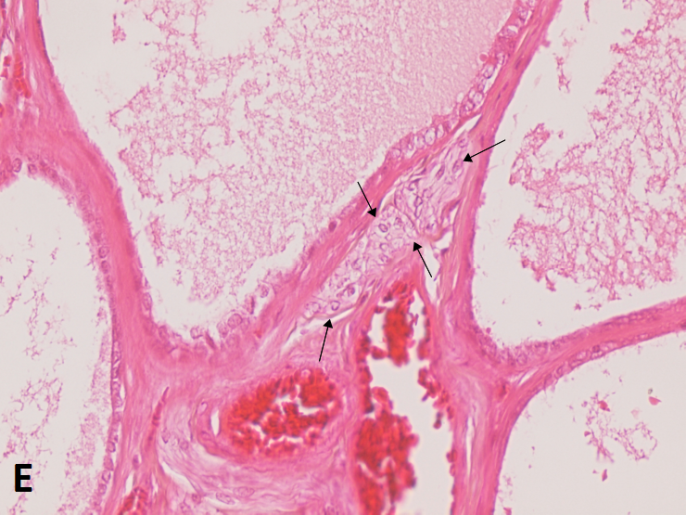


**
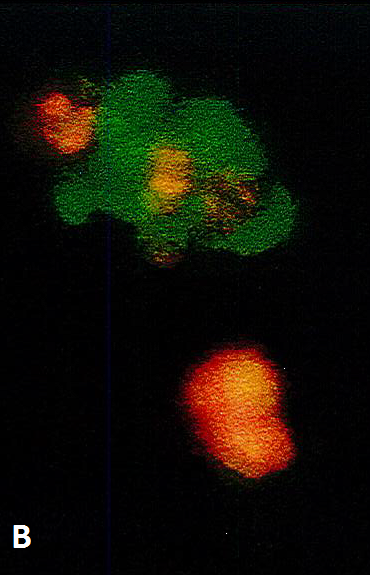

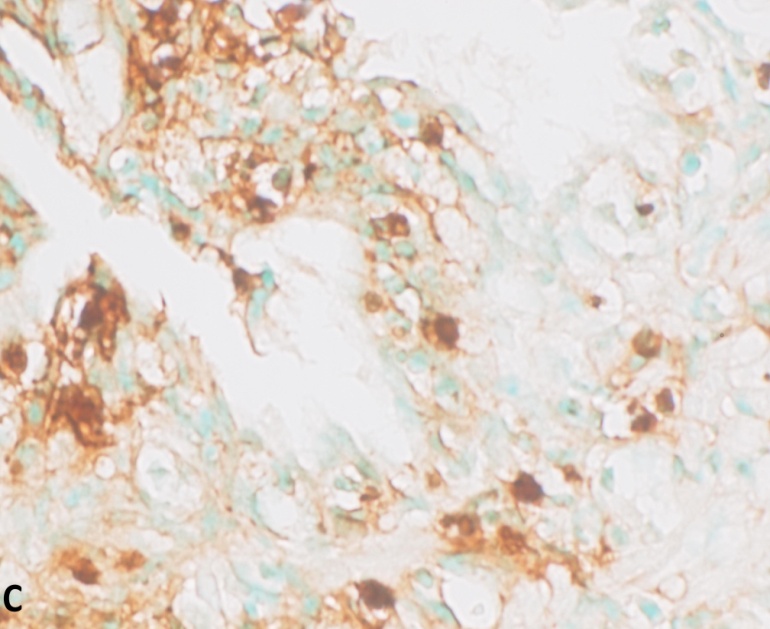

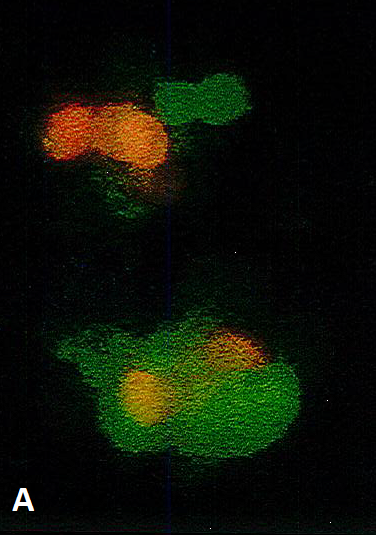
Figure 3**


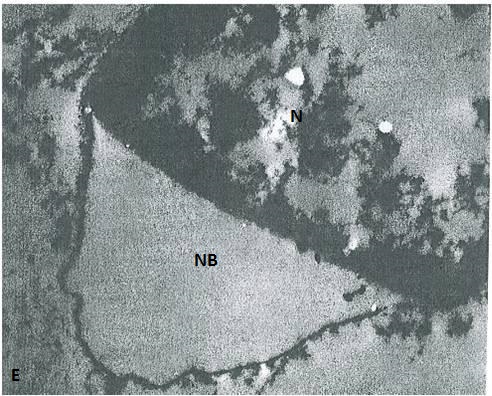


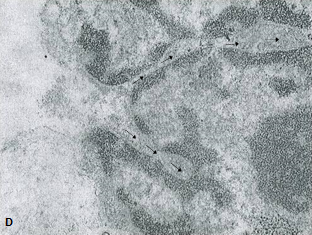

Supplement: Supplementary file 1 — Supplementary material 1 (DOCX 3763 kb) [file 345_2018_2185_MOESM1_ESM.docx]
